# Effects of Exercise Training in Patients with Lung Cancer during Chemotherapy Treatment

**DOI:** 10.21315/mjms2023.30.2.13

**Published:** 2023-04-18

**Authors:** Muheebur Rehman, Uzair Ahmad, Mehwish Waseem, Babar Ali, Muhammad Iqbal Tariq

**Affiliations:** 1 Department of Life Science, Abasyn University, Peshawar, Pakistan; 2 College of Physical Therapy, Northwest Institute of Health Sciences, Peshawar, Pakistan; 3 Faculty of Rehabilitation and Allied Health Sciences, Riphah International University, Islamabad, Pakistan; 4 Department of Paramedics, Medical Teaching Institution, Khyber Teaching Hospital, Peshawar, Pakistan

**Keywords:** lung neoplasms, exercise therapy, physical activity, radiation therapy, neoadjuvant

## Abstract

**Background:**

Cancer is the second greatest cause of death and disability after cardiovascular disease.

**Objective:**

To determine the effects of exercise training in patients with lung cancer during chemotherapy treatment.

**Methods:**

A randomised clinical trial was conducted in Shaukat Khanum Memorial Cancer Hospital and Institute of Radiotherapy and Nuclear Medicine (IRNUM) Peshawar. A total of 40 participants were randomly divided into two groups: i) the Experimental group (EG, *n* = 20) and ii) Control group (CG, *n* = 20). Both groups received exercise training for 4 weeks, with five sessions per week. The EG received pulmonary rehabilitation and aerobic training. The CG received only pulmonary rehabilitation. Both groups were evaluated at baseline and after 6 weeks through Mindful Attention Awareness Scale (MAAS) Urdu version, Six Minute Walk Test (6MWT), digital spirometry, Borgs scale, Hospital Anxiety and Depression Scale (HADs) and Visual Analogue Scale (VAS).

**Results:**

Both the EG and CG showed significant improvement in MAAS scores at post-study with a (*P* < 0.001). The scores of 6MWT were improved significantly in both groups after intervention with a (*P* = 0.001). The patient’s anxiety scores were significantly improved in both groups after intervention with a (*P* < 0.001), while depression scores were also improved considerably between the two groups at post-level with a (*P* < 0.001). Regarding spirometry value, both groups showed significant improvement after intervention for forced expiratory volume in 1 s (FEV1), forced vital capacity (FVC) and FEV1/FVC (*P* < 0.001). Both groups show significant differences in patient pain intensity and dyspnea at post-level with *P* < 0.001.

**Conclusion:**

This study concluded that pulmonary rehabilitation along with aerobic training can be more effective than pulmonary rehabilitation alone for patients with lung cancer during chemotherapy treatment.

## Introduction

After cardiovascular disease, the second greatest cause of disability and death is cancer. Each year more than 1.6 million new situations of lung cancer are detected around the world (1, 2). Cancer of the lung is considered to be the cause of death firstly in men and then secondly in women. The five Asian nations with the highest standardised lung cancer mortality rates were the Democratic Republic of Korea (40.9 per 100,000) followed by China (32.5%), Armenia (32%), Turkey (31%) and Timor-Leste (27.9%) (3). The research from 2020 states that the lifetime risk of lung cancer is roughly 1 in 55 for Malaysian men. The risk is highest for men of Chinese descent (1 in 43), then Malays (1 in 62) and Indians (1 in 103) (4). In Europe, the general 5-years the survival rate for cancer in lung patients is about 10%; survival among those who undergo surgery is 40% (5). Every year, around 150,000 new cases of cancer are reported in Pakistan, with a high mortality rate of 60%–80% (6). According to the Karachi Cancer Registry (KCR), the most frequent cancer among males is lung cancer, which is linked to cigarette smoking, followed by oral cancer, which is linked to excessive tobacco smoking and smokeless tobacco use (7). Lung cancer was the third most reported cancer in Pakistan in 2012, according to Global Cancer Database (GLOBOCON), while the Pakistan Health Research Council (PHRC) projected it to be the tenth most common disease in 2016 (8). The symptoms of probable lung cancer that patients have reported to their doctors have been found (9). Hemoptysis, cough, weariness, dyspnea, chest pain, weight loss, and anorexia were all listed as lung cancer symptoms in the National Institute for Health and Care Excellence (NICE) expected cancer referral recommendations (10). The two most frequent histological forms of primary lung cancer are small cell lung cancer (SCLC) and non-small cell lung cancer (NSCLC) (11).

Physical activity levels are frequently reduced, resulting in fitness loss and loss of exercise capacity, atrophy and weakness of muscle, and a drop in respiratory function and weakness in the breathing muscle (12, 13). Patients with lung cancer can engage in physical fitness activity safely at all stages of the disease and therapy (14, 15). Furthermore, there is evidence that physical fitness activity lowers the causes of various types of cancer (16) and the causes of cancer again chance by up to 40% (17). Patients may live longer, have fewer chronic diseases and have fewer cancer symptoms if they engage in physical activity (18). The American Thoracic Society and the European Respiratory Society claim that pulmonary rehabilitation increases exercise capacity and quality of life while reducing dyspnea (19). Chemotherapy is frequently combined with long-term usage of high-dose corticosteroids, which can lead to muscular atrophy and mitochondrial dysfunction (20). In addition, loss of skeletal muscle has been linked to higher toxicity (i.e. poor chemotherapy tolerance) and a poor prognosis (21). In patients with cancer, exercise physically counteracts the muscle strength loss experienced after treatment (22). High-intensity interval training induces significant muscle adaptation and health benefits in a time-efficient manner (23), which has been considered safe in cancer patients (24), including in the current trial during chemotherapy (25). The main aims of cancer rehabilitation are to improve the activity of daily living and to modify the patient’s lifestyle as an outcome of the condition and its management (26). Exercise fitness training in cancer patients is normally performed under supervision; for which screening of patient and assessment, proper exercises and modified activities are designated to make an individualised rehabilitation plan according to the stages of the disease, the type of treatment and motivation of the patient (27, 28).

This study is significant because it may aid in developing improved therapies that improve patient survival and quality of life. Research can ultimately raise the number of survivors who are still coping with the disease and offer a better and longer future for people diagnosed with lung cancer. This study has a significant multidisciplinary influence on the community since it promotes reducing inactive time throughout the entire cancer journey and understanding exercise safety standards. Empowering patients to take charge of self-care and providing such training which benefits the entire lung cancer community various ways. In addition, this study enables all medical and healthcare professionals to apply the findings to future healthcare facilities to increase community survival.

Most of research, meta-analyses and comprehensive reviews currently available are focused on surgical procedures and post-operative PR (pulmonary rehabilitation) in a patient with lung cancer. Gaps relating to the Control group (CG) have also been noted in the literature. The current study intended to close this gap by devising a systematic intervention strategy for both the CG and Experimental group (EG). This study may help community health workers become more aware of the importance of including an exercise training programme in the intervention plan for lung cancer patients, improving their survival and quality of life. It will also contribute to the literature on the lack of oncology rehab for patients who are solely getting chemotherapy. This study aimed to determine the effects of exercise training on patients having lung cancer undergoing chemotherapy treatment.

## Methods

### Study Setting and Subjects

A randomised control trial was conducted in Shaukat Khanum Memorial Cancer Hospital and Research Centre (SKMCH&RC) Peshawar, and the Institute of Radiotherapy and Nuclear Medicine (IRNUM) Peshawar from 28 May 2021 to 30 December 2021. The clinical trial has been registered at (https://clinicaltrials.gov/) (identification number: NCT05158530). After obtaining the written informed consent, patients with NSCLC were randomly allocated to EG and CG via the opaque sealed envelopes method. Patients were recruited through purposive sampling technique having the followings; both genders aged between 20 years old and 55 years old with stage 1 and stage 2 NSCLC, including adenocarcinoma and squamous cell carcinoma, patients who had a diagnosis within 6 weeks of enrollment confirmed by histology, patients undergoing lung cancer chemotherapy and able to complete the Six Minute Walk Test (6MWT) and having WHO physical fitness scores range from 0 to 1. The exclusion criteria were patients with a history of surgery and trauma, having a disease other than lung cancer, having uncontrolled hypertension or unstable coronary artery disease, severe osteoarthritis, bone or central nervous system metastases, haemoglobin of less than 10 g/dL, WBC less than 3,500 per microlitre of blood and a patient taking doxorubicin.

A total of 40 patients were randomly allocated into two groups: EG (*n* = 20) and CG (*n* = 20). The sample size was estimated based on a reference study (29) using OpenEpi tool, with confidence interval = 95%, power = 80%, mean (SD) of post-forced vital capacity (FVC) score = 95 (11) of EG and CG mean (SD) of post-FVC score = 80 (21).

### Description of the Intervention

A 4-week in-hospital exercise training programme along with chemotherapy was provided. Both parties agree to receive exercise training for 4 weeks with five sessions per week. Interventions were stopped in the following situations regarding cancer chemotherapy, 24 h after chemotherapy, the condition anaemia (haemoglobin 8 g/L), neutropenia (WBC count 0.5 109 cells/L), thrombocytopenia (platelet count 50 109 cells/L), complaint of nausea, vomiting, disturbances of orientation, fatigue, sight disturbances, weakness and pain in muscle or bone within the 24 h. The EG received pulmonary rehabilitation and aerobic training. The pulmonary rehabilitation protocols included deep breathing exercise (10 repetitions × 3 sets), postural drainage (10 min × 2 sets / day), incentive spirometry (10 repetitions × 3 sets), active ankle and hand pumping exercise (10 repetitions × 3 sets).

According to Frequency, Intensity, Time and Type (FITT) protocols, the supervised hospital-based ergometer cycling was performed for 30 min (including 5 min of warm-up and 5 min of cool-down), 5 days a week for 4 weeks at 40%–60% intensity. Target heart rate was calculated through the Karvonen formula. The CG received only pulmonary rehabilitation. All the sessions were delivered by a senior physical therapist, specialised in cardiopulmonary rehabilitation. All the sessions were carried out by the same therapist, who was unaware whether a participant was enrolled in the EG or the CG.

### Measures

The Visual Analogue Scale (VAS), 6MWT, Mindful Attention Awareness Scale (MASS) Urdu version, spirometer and Hospital Anxiety and Depression Scale (HADs) were used for the ‘pre-’ & ‘post-’ assessment of patients undoing chemotherapy during the study. The principal investigator in charge of the baseline and post-intervention evaluation had no idea whether a participant was in the EG or the CG. VAS was used to calculate the participant’s pain intensity before and after chemotherapy. A VAS is an evaluative tool that assess pain intensity. VAS is sufficiently reliable to assess acute pain (30). The 6MWT validly evaluates functional performance in people with chronic cardiopulmonary diseases that can be used across the disease spectrum (31). The 6MWT appears to be as valid and dependable in cancer patients as it is in healthy aged, cardiac and pulmonary patients. As a result, it can be suggested for cancer patients (32). The MASS assesses mindfulness characteristics consisting of 15-items. For scoring, calculate the average of the 15 things. The higher the score, the more dispositional mindfulness there is (33). The MAAS Urdu version scale has been found as an accurate and reasonable instrument to measure the level of mindfulness (34). Digital spirometer was used to calculate pulmonary function. Forced expiratory volume in 1 sec (FEV1), forced vital capacity (FVC) and a ratio of the two calculations (FEV1/FVC) were assessed by the guideline recommendations from the European Respiratory Society. The modified Borg scale is widely used in physiotherapy field to identify the workload during the training of muscles, and the clinical importance of the rehabilitation outcome (perceived dyspnea during physical activity) has been established (35). HADs was used to assess the participants’ anxiety and depression levels before and after chemotherapy treatment. HADs is a self-rating scale that assess anxiety and depression in hospitals and community settings. A score of 0–7 is regarded as normal, 8–10 is borderline abnormal and 11–12 considered as abnormal (36).

### Statistical Analysis

All the data were analysed by IBM SPSS version 25.0. When the normality test was applied using the Shapiro-Wilk test, it shows some variables were normally distributed, and other were not normally distributed. Parametric test, i.e. independent *t*-test, was applied on normally distributed data for between-group analysis, and paired *t*-test was applied for within-group analysis. In case of no normal distribution, the Mann-Whitney U test was used for between-group analysis and the Wilcoxon test was applied for within-group analysis.

## Results

**Figure 1:**
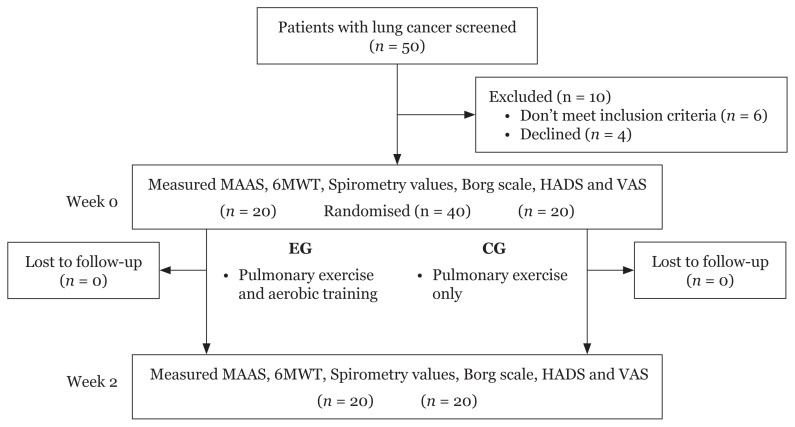
Consort diagram

The randomised clinical trial was conducted at Shaukat Khanum Memorial Cancer Hospital and Research Centre Peshawar and the Institute of Nuclear Medicine and Radiotherapy (IRNUM) Peshawar. Participants were randomly distributed into two main groups: EG and CG. A total of 40 participants were included in the study, of which 20 participants (50%) were allocated to the EG and 20 participants (50%) were allocated to the CG. Out of the total 40 participants, 24 (58.5%) were male and 16 (39%) were female. The EG received pulmonary rehabilitation and aerobic training, whereas the CG received pulmonary rehabilitation-only (Figure 1). The EG had a mean age of 48.15 (SD = 4.0) years old, while the CG had a mean age of 48.30 (SD = 3.8) years old. The BMI score of the EG was 23.68 (SD = 1.6) and the BMI score of the CG was 22.5 (SD = 2.2). Most patients were diagnosed with adenocarcinoma with a frequency of 25 (61%). Most patients were in stage I tumours with frequency ranges of 30 (73%). According to smoking history, 27 (65%) patients were former smokers (Table 1).

Before the intervention, a between-group analysis revealed no significant difference between the groups on all variables (*P* > 0.001). After the intervention, there is a statistical difference in MAAS scores between the two groups with a *P* < 0.001 (mean difference (MD) = 0.36; 95% CI: 0.19, 0.54) (Table 2).

Similarly, the 6MWT scores of the two groups differ statistically after the intervention with a *P* = 0.001 (MD = 47.4; 95% CI: 20.6, 74.3) (Table 2).

The patient’s anxiety scores were statistically different between groups after intervention with a *P* < 0.001 (MD = −4.4; 95% CI: −5.39, 3.42) while, the depression scores were also significantly different between the two groups at post-level with a *P* < 0.001 (MD = −5.3; 95% CI: −6.49, −4.14) (Table 2).

Regarding spirometry value, there is a statistical difference in spirometry values between the groups after the intervention for FEV1 with a *P* < 0.001 (MD = 10.2; 95% CI: 7.93, 12.5), for FVC with a *P* < 0.001 (MD = 10.0; 95% CI: 7.69, 12.3) and for FEV1/FVC with a *P* < 0.001 (MD = 10.5; 95% CI: 8.00, 13.01) (Table 2).

The scores of the Borg scale and pain intensity were significantly different between both groups after the intervention (*P* < 0.001) (Table 5).

Within-group analysis of the EG shows that all studied factors are statistically significant at pre-test and post-test levels (*P* < 0.001), as mentioned in (Table 3).

Within-group analysis of the CG shows that all studied factors are statistically significant at pre-test and post-test levels (*P* < 0.001) as mentioned in (Table 4).

## Discussion

The study’s objective was to determine the effects of exercise training in patients with lung cancer undergoing chemotherapy treatment. The current study’s results suggested a statistical difference between the EG and CG regarding MAAS, 6MWT, spirometry values, Borgs scale, HADs and VAS.

Our study found a significant difference in mindfulness (MAAS score) between groups. The mean value of the MAAS score was significantly increased in EG than in CG. These findings are supported by Mothes et al. (37), who assessed whether aerobic exercise increases mindfulness. A random allocation of 149 subjects in the EG and CG who had undergone 12-week training. The author observed increases in mindfulness occurred in a group that received aerobic exercise training. At the same time, in current study the EG also received the aerobic exercise along with pulmonary rehabilitation. A moderate correlation was found between rising dispositional mindfulness and improved mental health. This study demonstrates for the first time how regular aerobic exercise can improve dispositional mindfulness. Future studies required to determine the best way to apply aerobic exercise’s ability to improve mindfulness.

Our study observed significant differences in 6MWT between groups. The mean value of the 6MWT score was significantly increased in EG than in CG. The 6MWT scores of the control group had slightly changed. These findings are supported by Dhillon et al. (38) conducted a randomised control trial on 112 patients to find the effect of physical activity in patients with advanced lung cancer. A 2-month of physical activity intervention did not improve quality of life and fatigue. However, from baseline to 2 months, both groups doubled the mean distance in the 6MWT, which they maintained at 4 months and 6 months. Clinically significant improvements in populations with chronic obstructive pulmonary disease are those of 30 m. In our study the 65 m difference was observed in the EG. The author may not have included the most advanced cases of lung cancer and the intervention may not have been structured enough to distinguish between groups in terms of physical activity. Jastrzebski et al. (39) conducted a randomised control trial on 20 patients to find the effect of rehabilitation in patients with advanced level chemotherapy lung cancer. An 8-week pulmonary rehabilitation tends to increase the 6MW distance in a patient with lung cancer. However, this study has several limitations. The number of lung cancer patients in the sample was small. The daily sessions for rigorous fitness regimen could last up to 2 h. Furthermore, the study was conducted in an academic medical facility, so results cannot be applied to patients with advanced lung cancer who seek oncology care in the community. Our study results also correspond to Licker et al. (40), which assessed the impact of interval training with high intensity on 151 pre-operative patients with lung cancer. The study revealed a significant improvement in aerobic performance measured by 6MWT.

The current study found that mean value of anxiety and depression was significantly improved in the EG than in the CG. Little changes were observed in the CG. These findings were supported by Molassiotis et al. (41) conducted RCT on 46 patients to find the impact of inspiratory muscle training in the lung cancer population. A 12-week inspiratory muscle training (IMT) was given to EG and the psychological distress measured via HADs. The author observed changes in HADs scores in a group that followed inspiratory muscle training. However, at baseline, half of the patients in the IMT group reported feeling exhausted after the IMT training. Although this study decided not to analyse the self-reported data on oxygen, opioid and steroid use because there was a significant amount of missing information, it is acknowledged that these are important factors in a future larger trial, both in terms of medication use and as variables that require stratification. Dhillon et al. (38), found the effect of physical activity in patients with advanced lung cancer. This study investigated feelings of anxiety and depression using the Anxiety/Depression General Health Questionnaire. This study found no statistical difference in feelings of anxiety and depression between the intervention group and the CG on completion of the 8-week intervention. These findings are inconsistent in literatures due the variations of measuring tools, type and length of exercise training. Further studies should investigate these variables.

The mean value of FEV1, FVC and FEV1/FVC score measured through spirometer was significantly improved in EG than CG. Morano et al. (42) investigated the effect of 4-week pulmonary rehabilitation versus chest physiotherapy on 24 patients undergoing lung cancer resection. The study found a significant improvement in FVC while noticed no improvement in FEV1. The limited sample size and patient drop out were two extremely important drawbacks of this study that raise questions regarding its generalisability. These results need to be confirmed by additional research, especially on a large scale with well design study. Another study by Rutkowska et al. (29) randomised 40 patients, to find the effect of a 4-week physical training exercise in a patient with non-small types of lung cancer undergoing the chemotherapy session. The study found more significantly improved values of spirometry (FEV1, FVC and FEV1/FVC) in the EG.

A retrospective study by Tarumi et al. (43) found changes in pulmonary function when assessed peri-operative intensive pulmonary rehabilitation programme (PPRP) for 10 weeks in 82 patients with a non-small cell IIB-IV stage lung cancer following chemotherapy session. The author found a significant increase in FEV1 and FVC. Because this study was conducted over a long period of time, questions have been raised about the consistency of the PPRP techniques. These results should be carefully interpreted because there was no CG in the current investigation. One of the study’s weakness is the absence of an analysis of additional characteristics that affect surgical tolerance, such as the 6-min walking distance and the peak oxygen intake. Future research assessing these variables is required.

The VAS scores were significantly improved in the EG. The mean value of pain intensity was improved considerably more in the EG than in the CG. These findings were observed by Jeong et al. (44) who investigated the effect of pulmonary rehabilitation education on the caregiver of lung cancer patients who underwent lung resection. This study randomly allocated 22 patients to an EG while 19 to a CG. A 4-week pulmonary rehabilitation education programme was administered to the EG. A significant decrease in VAS score was observed in the EG.

The scores on the Borg scale were significantly improved in the EG. The mean value of the Borg scale was significantly improved in the EG than in the CG. Hwang et al. (45) randomly allocated 24 patients with NSCLC (stages IIIa–IV) to either an EG or a CG. The EG received cycling ergometer or treadmill training performed thrice a week for 24 sessions for 30 min–40 min. The authors reported a significant decrease in dyspnea. However, the current research examining exercise training for people with lung cancer has identified heterogeneity. More researches should be done to confirm the exercise advantages and to elucidate the underlying mechanisms because focused therapy improves survival rates. Rutkowska et al. (29) investigated the effect of 4-week exercise training on inpatients with NSCLC undergoing the chemotherapy treatment session. Twenty patients were randomly allocated to the EG and 10 in the CG. The study observed a significant improvement in dyspnea using the Borg scale in the EG (29).

## Conclusion

This study concluded that pulmonary rehabilitation along with aerobic training can be more effective than pulmonary rehabilitation alone for patients with lung cancer during chemotherapy treatment. Pulmonary rehabilitation along with aerobic training can improve the mindfulness, walking distance, lung function, dyspnea and pain of lung cancer patients. Furthermore, this study reduced anxiety and depression in lung cancer patients.

### Study Limitations

The limitation includes the small number of participants enrolled and the lack of biochemical markers measurements in the EG compared with the CG. The small sample size was one of the study’s limitations. A multicentre study with a large sample size should be conducted to confirm our results. Future studies could also focus on the individual exercise training programme to target the different phases of lung cancer.

### Recommendations

A multi-centred study should address the different stages and various outcomes of lung cancer. Such studies may be helpful and extend the role of a rehabilitation programme to be part of the intervention in managing lung cancer.

## Figures and Tables

**Table 1:** Patient demographics (*n* = 40)

Variables	EG (*n* = 20) *n* (%)	CG (*n* = 20) *n* (%)
Age (years old)^a^	48.1 (4.0)	48.3 (3.8)
Height (cm) a	166.1 (3.8)	166.5 (3.4)
Weight (kg) a	65.4 (5.8)	62.7 (7.4)
BMI (kg/m^2^) a	23.6 (1.6)	22.5 (2.2)
Gender		
Male	11 (52.4)	13 (61.9)
Female	9 (42.9)	7 (33.3)
Diagnosis		
Adenocarcinoma	12 (57.1)	13 (61.9)
Squamous cell carcinoma	8 (38.1)	7 (33.3)
Stage		
I	16 (76.2)	14 (66.7)
II	4 (19)	6 (28.6)
Smoking history		
Current	0	0
Former	14 (66.7)	13 (61.9)
Never	6 (28.6)	7 (33.3)
WHO grading		
Grade 0	7 (33.3)	8 (38.1)
Grade I	13 (61.9)	12 (57.1)

Note:

a mean (SD)

**Table 2:** Comparison of variables between experimental and control groups

Variables	Mean (SD) between group									

	Pre-test					Post-test				
	
	EG (*n* = 20)	CG (*n* = 20)	Mean difference (95% CI)	*t*-statistic (df)	*P*-value^a^	EG (*n* = 20)	CG (*n* = 20)	Mean difference (95% CI)	*t*-statistic (df)	*P*-value^a^
MAAS	4.41 (0.4)	4.48 (0.3)	−0.07 (−0.32, 0.17)	−0.58 (37)	0.56	5.34 (0.2)	4.98 (0.2)	0.36 (0.19, 0.54)	4.21 (37)	*P* < 0.001
6MWT	468.31 (49.4)	476.40 (45.9)	−8.08 (−38.6, 22.4)	−0.53 (38)	0.59	533.58 (43.5)	486.10 (40.2)	47.4 (20.6, 74.3)	3.58 (38)	*P* = 0.001
HADs anxiety (0–21)	13.63 (1.4)	13.39 (1.4)	0.23 (−0.66, 1.14)	0.52 (38)	0.60	4.23 (1.5)	8.64 (1.5)	−4.4 (−5.39, 3.42)	−9.03 (36)	*P* < 0.001
HADs depression (0–21*)*	12.75 (2.2)	13.02 (2.0)	−0.27 (−1.65, 1.10)	−0.40 (38)	0.69	4.10 (1.7)	9.42 (1.9)	−5.3 (−6.49, −4.14)	−9.14 (38)	*P* < 0.001
FEV1	73.64 (2.7)	73.10 (3.8)	0.54 (−1.5, 2.67)	0.51 (38)	0.609	87.08 (3.3)	76.86 (3.6)	10.2 (7.93, 12.5)	9.04 (36)	*P* < 0.001
FVC	76.22 (2.3)	74.82 (3.4)	1.4 (−0.50, 3.31)	1.4 (33)	0.142	87.83 (3.4)	77.82 (3.6)	10.0 (7.69, 12.3)	8.76 (36)	*P* < 0.001
FEV1/FVC	64.99 (2.8)	66.28 (3.4)	−1.2 (−3.34, 0.76)	−1.2 (37)	0.212	80.77 (4.2)	70.26 (3.5)	10.5 (8.00, 13.01)	8.48 (38)	*P* < 0.001

Notes: MAAS = Mindful Attention Awareness Scale; 6MWT = Six Minute Walk Test; HADs = Hospital Anxiety and Depression scale; FEV1 = Force Expiratory Volume in 1 sec; FVC = Force Vital Capacity; FEV1/FVC = ratio;

a Independent *t*-test;

EG = Experimental group; CG = Control group

**Table 3:** Comparison of variables pre- and post within EG

Variables	EG (*n* = 20)				

	Mean (SD)		Mean difference (95% CI)	*t*-statistic (df)	*P*-value^a^

	Pre-test	Post-test			
MAAS	4.42 (0.4)	5.34 (0.2)	−0.92 (−1.07, −0.76)	−12.1 (18)	*P* < 0.001
6MWT	468.31 (49.4)	533.58 (43.5)	−65.2 (−71.4, −59.1)	−22.1 (19)	*P* < 0.001
HADs Anxiety (0–21)	13.63 (1.4)	4.23 (1.5)	9.39 (8.40, 10.3)	19.9 (19)	*P* < 0.001
Depression (0–21)	12.75 (2.2)	4.10 (1.7)	8.65 (7.72, 9.57)	19.5 (19)	*P* < 0.001
FEV1	73.69 (2.8)	87.08 (3.3)	−13.39 (−15.6, −11.1)	−12.5 (18)	*P* < 0.001
FVC	76.03 (2.2)	87.83 (3.4)	−11.80 (−13.2, −10.3)	−16.6 (18)	*P* < 0.001
FEV1/FVC	64.99 (2.8)	80.29 (3.7)	−15.29 (−17.6, −12.9)	−13.4 (18)	*P* < 0.001

Note:

a Paired *t*-test

**Table 4:** Comparison of variables pre- and post within CG

Variables	CG (*n* = 20)				

	Mean (SD)		Mean difference (95% CI)	*t*-statistic (df)	*P*-value^a^

	Pre-test	Post-test			
MAAS	4.48 (0.3)	4.95 (0.2)	−0.473 (−0.57, −0.37)	−10.16 (18)	*P* < 0.001
6MWT	476.40 (45.9)	486.10 (40.2)	−9.70 (−14.6, −4.72)	−4.07 (19)	*P* = 0.001
HADs Anxiety (0–21)	13.31 (1.4)	8.64 (1.5)	4.66 ( 4.06, 5.26)	16.42 (18)	*P* < 0.001
Depression (0–21)	13.02 (2.0)	9.42 (1.9)	3.60 ( 2.86, 4.34)	10.19 (19)	*P* < 0.001
FEV1	73.10 (3.8)	76.86 (3.6)	−3.75 ( −4.26, −3.25)	−15.56 (19)	*P* < 0.001
FVC	74.82 (3.4)	77.82 (3.6)	−3.00 ( −3.92, −2.08)	−6.82 (19)	*P* < 0.001
FEV1/FVC	66.28 (3.4)	70.26 (3.5)	−3.98 ( −4.63, −3.33)	−12.77 (19)	*P* < 0.001

Note:

a Paired *t*-test

**Table 5:** Comparison of variables between experimental and control groups

Outcome	Median (IQR) within groups							Median (IQR) between groups				
	
	EG (*n* = 20)			CG (*n* = 20)			Pre-test			Post-test		
			
	Pre-test	Post-test	*P*-value^a^	Pre-test	Post-test	*P*-value^a^	EG	CG	*P*-value^b^	EG	CG	*P*-value^b^
VAS (0–10)	6.16 (1.7)	2.17 (0.6)	< 0.001	6.16 (1.7)	3.92 (0.8)	< 0.001	6.16 (1.7)	6.16 (1.7)	1.000	2.17 (0.6)	3.92 (0.8)	< 0.001
Borg scale	2.01 (1.4)	0.19 (0.8)	< 0.001	2.01 (0.8)	1.87 (0.8)	< 0.001	2.01 (1.4)	2.01 (0.8)	0.97	0.19 (0.8)	1.87 (0.8)	< 0.001

Notes: VAS = Visual Analogue Scale; EG = Experimental group; CG= Control group;

a Wilcoxon test;

b Mann-Whitney U test
